# Rebound Hypercalcemia After Denosumab Cessation in Adult Fibrous Dysplasia: A Case Report and Clinical Alert

**DOI:** 10.1155/crie/4553039

**Published:** 2025-09-04

**Authors:** Danni Liu, Jiayi Chen, Hongmei Chen, Wenxue Hu, Jinxin Lin

**Affiliations:** ^1^Department of Nephrology, Guangdong Provincial People's Hospital, Guangdong Academy of Medical Sciences, Guangdong Provincial Geriatrics Institute, Southern Medical University, Guangzhou, China; ^2^Department of Endocrinology, Guangdong Provincial People's Hospital, Guangdong Academy of Medical Sciences, Guangdong Provincial Geriatrics Institute, Southern Medical University, Guangzhou, China

**Keywords:** adult patient, denosumab, fibrous dysplasia, rebound hypercalcemia

## Abstract

**Background:** Fibrous dysplasia (FD) is a rare congenital bone disease. Denosumab, a monoclonal antibody targeting nuclear factor kappa-B ligand (RANKL), suppresses osteoclast activity and exhibits therapeutic potential for FD.

**Case Presentation:** We present the case of an adult female patient diagnosed with FD who had undergone 7 treatment cycles of denosumab (120 mg/dose, sc.) with a cumulative dose of 840 mg. After discontinuing denosumab for 7 months, the patient experienced a crisis of rebound hypercalcemia.

**Conclusion:** Although, rare reports of hypercalcemia induced by discontinuation of denosumab are primarily seen in adolescents. By reporting this case, we aim to alert clinicians to the risk of rebound hypercalcemia in adult patients with FD undergoing denosumab treatment.

## 1. Background

Fibrous dysplasia (FD), a rare congenital skeletal disorder, originates from postzygotic gain-of-function mutations in GNAS (G protein α-subunit). This molecular defect drives pathological receptor activator of nuclear factor kappa-B ligand (RANKL) overexpression within FD lesions. Gene mutation induces persistent adenylyl cyclase activation, resulting in elevated cyclic adenosine monophosphate (cAMP) concentrations. This heightened cAMP activates downstream signaling pathways, leading to the pathological displacement of normal bone with fibrous tissue and immature woven bone [1]. RANKL is a cell surface protein involved in numerous cellular processes, including osteoclast formation. Studies have found that RANKL is stably expressed in lesional tissues of FD patients and in in vitro cultured FD bone marrow mesenchymal stem cells. Moreover, FD patients exhibit substantially increased circulating RANKL concentrations, which demonstrate a strong correlation with clinical disease severity [2]. As a monoclonal antibody targeting RANKL, denosumab inhibits osteoclast-mediated bone resorption, thereby preventing bone loss and reducing fracture risk [3]. In FD patients, denosumab has demonstrated certain clinical efficacy. It can reduce lesional activity, promote osteoblast maturation and bone formation, while concurrently lowering serum calcium levels [4]. However, it is noteworthy that there has been an increasing number of case reports describing rebound hypercalcemia following denosumab discontinuation in recent years [5]. Although this complication is extremely rare, its severe consequences warrant the attention of clinicians. While this complication has been predominantly observed in adolescent patients, we report a case of hypercalcemia induced by denosumab in an adult female FD patient.

## 2. Case Presentation

**Figure 1:**
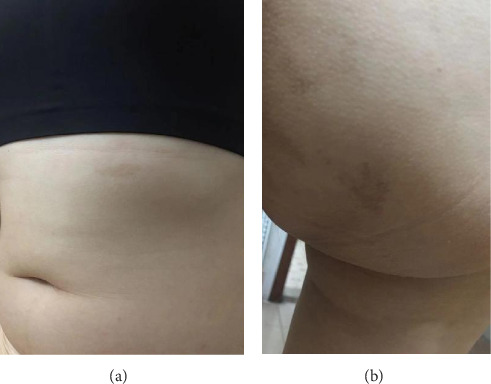
Atypical café-au-lait spots: (A) left upper abdominal quadrant (3 cm in length; 0.5 cm in width) and (B) right gluteal region (2 cm in length; 1 cm in width).

**Figure 2:**
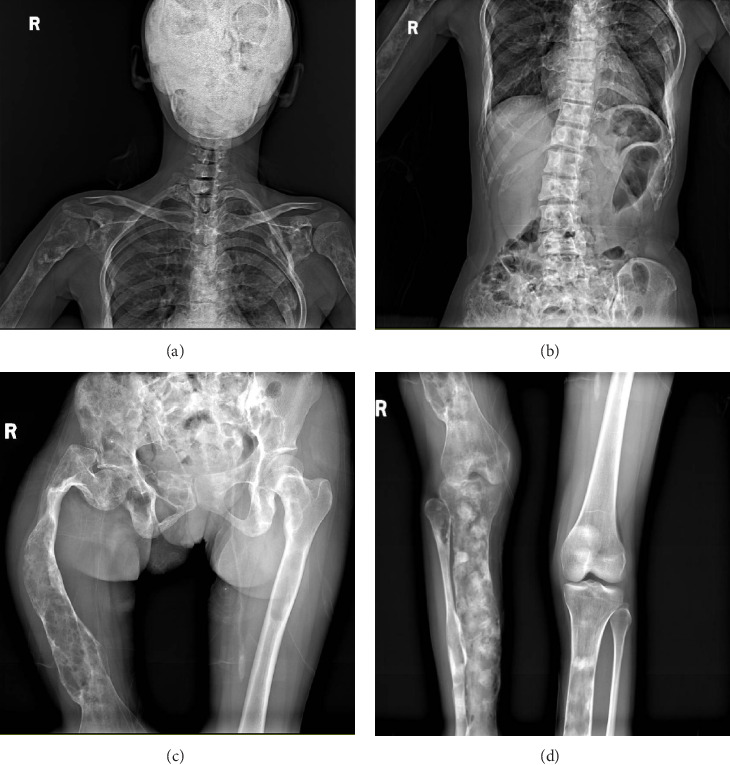
Radiographic examination at presentation revealed: (A) multiple osteolytic lesions in the bilateral ribs, scapulae, and humeri, (B) scoliosis with osteolytic lesions in the thoracolumbar spine, (C) classic “Shepherd's crook” deformity in the right proximal femur with osteolytic lesions in bilateral femora, and (D) osteolytic lesions in the bilateral tibiae and right fibula.

**Figure 3:**
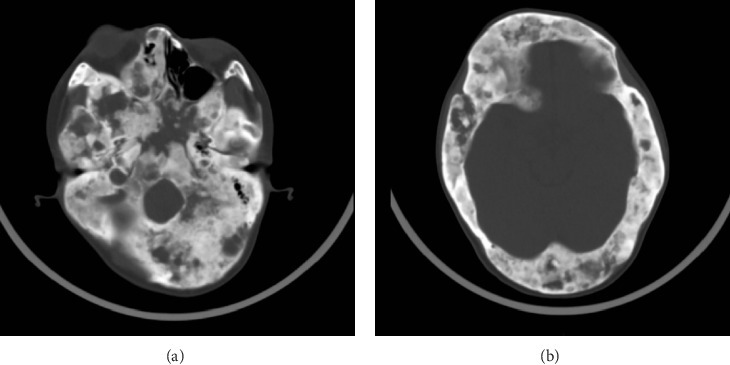
Cranial CT revealed: (A) bony lesions in the skull base and (B) bony lesions in the calvarium.

**Figure 4:**
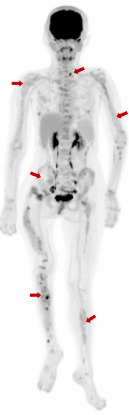
Whole-body 18 F-FDG PET/CT scan of the patient demonstrated diffuse tracer uptake (red arrows) in fibrous dysplasia lesions involving the spine, pelvis, bilateral extremities, and among other sites.

The patient, a 32-year-old female, presented with a temperature of 37°C, pulse rate of 102 beats/min, respiratory rate of 20 breaths/min, blood pressure of 121/101 mmHg, weight of 33 kg, and height of 146 cm. The patient exhibited developmental abnormalities and malnutrition. Craniofacial anomalies were noted, with bony prominences visible on the right forehead. Café-au-lait spots were observed in the right gluteal regions and left thoracic (Figure 1). The patient had spinal curvature deformities, with thoracolumbar scoliosis, asymmetrical lower limbs, and unequal leg lengths. Imaging revealed increased thickness and uneven density of the right zygomatic bone, as well as generalized density increase in the right maxillary sinus. The cervical spine showed no significant scoliosis, and the thoracolumbar spine exhibited mild reverse S-shaped curves. Multiple cystic low-density lesions with patchy high-density areas were seen in the vertebral bodies, pelvis, bilateral femurs, ribs, scapulae, humeri and cranial bone (Figures 2–4). Genetic testing revealed no copy number variations or methylation mutations near the GNAS gene. Molecular genetic testing of regions encoding skeletal development-related genes on the human genome revealed no single nucleotide variations, small fragment insertions or deletions, or large-scale copy number variations and repeat sequence abnormalities. The information above has been obtained with the patient's informed consent signed.

**Figure 5:**
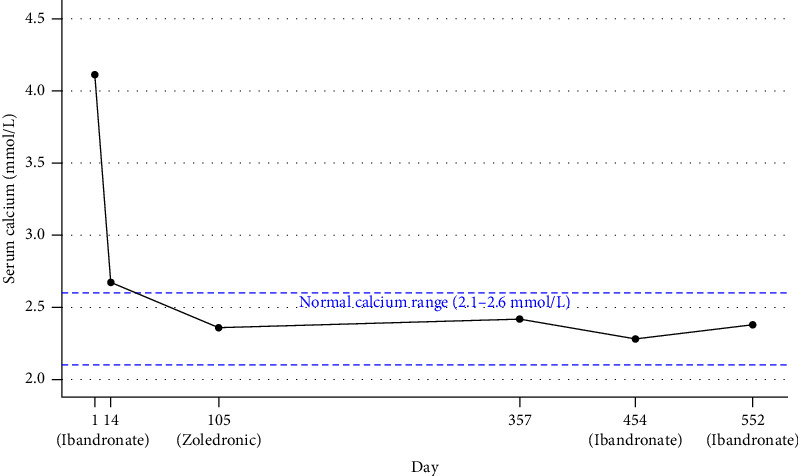
Changes in serum calcium levels following bisphosphonate treatment.

The patient had a history of multiple fractures and was treated with denosumab injection for a right humerus fracture began on July 27, 2022, with subcutaneous injections of 120 mg per dose. The last injection was administered on March 8, 2023, completing 7 treatment cycles with intervals of 1 month for the first 6 doses and 2 months for the seventh dose, with a cumulative dose of 840 mg. Following completion of the treatment course, the patient experienced notable bone pain. Seven months after the last denosumab injection, the patient presented with a triad of symptoms: emesis, appetite suppression, and polyarthralgia. Laboratory analysis detected serum calcium at 3.75 mmol/L. Antiemetics, calcitonin, nutritional supplementation, diuresis and intravenous fluid therapy were initiated but showed poor efficacy. The patient sought endocrinology consultation on November 9, 2023, presenting serum calcium of 3.99 mmol/L. Treatment with salmon calcitonin and intravenous fluid therapy was initiated with limited improvement. Further investigations during hospitalization ruled out primary hyperparathyroidism, secondary hyperparathyroidism, and malignant tumors causing hypercalcemia, with discontinuation of denosumab considered the likely cause. Laboratory tests upon admission revealed hypercalcemia (serum calcium: 4.11 mmol/L) with concomitant hypokalemia (serum potassium: 2.12 mmol/L). Treatment included combined intravenous and oral potassium replacement, fluid resuscitation, and salmon calcitonin (100 IU/day) for calcium reduction. During hospitalization, ibandronate (2 mg) was added for calcium control. The patient was discharged following symptomatic improvement. Postdischarge monitoring showed mild fluctuations in serum calcium levels between 2.7 and 2.9 mmol/L. On February 18, 2024, the patient returned for follow-up, and the treatment regimen was adjusted to zoledronic acid (5 mg). The patient was discharged after normalization of serum calcium. Following regular follow-up, maintenance therapy with ibandronate (2 mg every 3 months) was initiated during a return visit on February 5, 2025. Serum calcium levels remained well-controlled throughout this treatment period (Figure 5).

## 3. Discussion

FD may involve a single bone (monostotic) or multiple bones (polyostotic), when associated with café-au-lait spots and hyperfunctioning endocrinopathies, it is classically termed McCune-Albright syndrome [6].This patient had a previous diagnosis of McCune-Albright Syndrome. However, upon admission during adulthood, further evaluation revealed that precocious puberty could not be definitively established. Additionally, the café-au-lait spots on the patient's left flank and right buttock were atypical. Based on these findings, FD was established as the definitive diagnosis. Hypercalcemia was suspected to be caused by cessation of denosumab treatment, after primary and secondary hyperparathyroidism and malignancy-induced hypercalcemia were ruled out. FD can be managed with either drug therapy or surgical intervention. Current management of FD encompasses both pharmacological and surgical approaches. Pharmacological therapy primarily provides symptomatic and supportive management; while bisphosphonates effectively alleviate FD-associated bone pain, they do not confer significant benefits for overall clinical outcomes or long-term prognosis [7, 8]. Surgery is generally indicated for correcting skeletal deformities and addressing pathological fractures, yet demonstrates limited efficacy in cases with extensive or severe disease involvement.

Denosumab is approved for treating osteoporosis and bone metastasis in adults. Nevertheless, recent studies demonstrate its significant efficacy in inhibiting disease progression across multiple disorders affecting immature bone development, including FD, central giant cell granuloma (CGCG), osteogenesis imperfecta, giant cell tumor of bone (GCTB), and aneurysmal bone cyst (ABC). Unexpectedly, these clinical studies also identified a withdrawal reaction previously unrecognized—hypercalcemia [5]. This phenomenon may arise because RANKL stimulates osteoclasts to differentiate into smaller, motile cells termed osteomorphs, which retain their fusion potential to form functional osteoclast and primarily localize to bone marrow and peripheral blood. Osteoclasts undergo cellular recycling through osteomorphs [9]. However, denosumab, by inhibiting RANKL, blocks this cellular recycling process, leading to the accumulation of osteomorphs. Cessation of denosumab therapy triggers accelerated fusion of osteomorphs into metabolically active osteoclasts, precipitating a sharp rise in bone degradation and subsequent systemic calcium mobilization [10]. Additionally, studies suggest that baseline bone turnover rate and calcium reservoir capacity may determine the severity of hypercalcemia following denosumab discontinuation. Notably, males typically exhibit rates compared to adults. Consequently, this phenomenon shows a potential male predominance in adolescent populations. Furthermore, the timing of hypercalcemia onset post-denosumab cessation varies across age groups: pediatric and adolescent patients frequently develop hypercalcemia within 3 months after discontinuation, whereas adults typically experience it beyond 3 months. The hypothesis also implies that discontinuation of extended denosumab regimens can induce rebound hypercalcemia in individuals with established skeletal maturity [5]. However, rebound hypercalcemia is rare in adults, potentially due to underrecognition of asymptomatic cases. Notably, this discontinuation reaction following a high-dose denosumab regimen has also been observed in the FD mouse model. Nonetheless, the study maintains that FD patients should initiate denosumab therapy early, with the aim of promoting disease reversal and achieving optimal therapeutic outcomes. Therefore, a rigorous evaluation of denosumab treatment strategies is of significant clinical importance [11]. During follow-up, bone turnover markers should be monitored. Meanwhile, Na[18F]F PET-CT also proved effective in assessing treatment-induced changes in bone turnover within the lesions following denosumab administration [12].

Furthermore, our summary of FD patients treated with denosumab suggests that a low-dose regimen incorporating progressively extended dosing intervals prior to discontinuation is associated with a lower incidence of rebound hypercalcemia or only mild, asymptomatic hypercalcemia in adults. In contrast, the present case, involving abrupt cessation of high-dose denosumab without prior interval extension, likely contributed to the development of severe rebound hypercalcemia. In pediatric cases [13–15], some studies have explored strategies including dose adjustment, shortening of dosing intervals during therapy, and prophylactic bisphosphonate use. However, robust evidence remains limited due to small cohort sizes. Collectively, these observations indicate that shortening dosing intervals during the active treatment phase combined with strategic extension of intervals prior to discontinuation may better leverage the drug's cumulative effect, potentially reducing hypercalcemia risk. For rebound hypercalcemia that has already occurred, there are currently no established treatment recommendations. Clinically, first-line interventions for this condition typically comprise intravenous hydration, diuretic administration, calcitonin, corticosteroids, bisphosphonates, and/or denosumab redosing. Current research supports the clinical utility of combined intravenous fluid resuscitation, diuretic therapy, corticosteroid administration, and nitrogen-containing bisphosphonates (pamidronate or zoledronic acid) for managing aneurysmal bone cysts (ABCs) in pediatric patients. Additionally, extended half-life bisphosphonates demonstrate superior efficacy in mitigating recurrent hypercalcemia episodes [16]. When managing the hypercalcemia crisis in this case of adult patient, we combined intravenous fluid resuscitation and calcitonin administration, which effectively improved the serum calcium levels. After discharge, the patient intermittently took ibandronate sodium and monitored serum calcium levels, with no further recurrence of hypercalcemia. 3 months later during a follow-up visit, treatment shifted to zoledronic acid (a bisphosphonate with a half-life of 146 h), resulting in normalized serum calcium levels and subsequent discharge. Upon reevaluation 3 months later, the patient's serum calcium levels remained normal, and zoledronic acid was temporarily continued.

A PubMed search was conducted to retrieve relevant studies on denosumab's therapeutic application in FD patients (Table 1). A review of 90 cases of FD treated with denosumab showed that most patients could effectively benefit from disease control after treatment with denosumab. Majoor demonstrated that the standard osteoporosis regimen (60 mg denosumab every 6 months) failed to achieve sustained suppression of bone turnover markers in FD patients. Expanding on these findings, Meier proposed an intensified 60 mg quarterly dosing schedule. Furthermore, he identified pretreatment bone turnover levels and posttreatment skeletal improvement as primary determinants of rebound phenomena, outweighing treatment duration or cumulative dose.

Rebound hypercalcemia following denosumab discontinuation is exceedingly rare, with a significantly higher proportion of reported cases occurring in pediatric patients compared to adults. This rarity primarily stems from the limited clinical experience with denosumab in FD management and its current exclusion from clinical guidelines. Consequently, large-scale clinical investigations are urgently needed to comprehensively assess the therapeutic outcomes and safety profile of denosumab in FD management Moreover, in adult patients, denosumab withdrawal-associated rebound hypercalcemia is characterized by a prolonged latent period and an exceedingly low incidence, which may contribute to insufficient clinical follow-up duration. Furthermore, patients who have used denosumab only seek medical attention when severe hypercalcemia and resulting discomfort occur, potentially leading to delayed detection of asymptomatic or mildly symptomatic hypercalcemia patients. Therefore, this case report aims to highlight the necessity for extended clinical follow-up in denosumab-treated adult cohorts, with biochemical monitoring every 2 months both during denosumab treatment and post-cessation. Healthcare providers should instruct patients and families on hypercalcemic symptom recognition, promoting early intervention and mitigating treatment complications [30]. For patients about to undergo denosumab treatment, practical preventive medication plans should be established.

However, this study has limitations. The patient tested negative for GNAS mutations, and post-denosumab treatment imaging data were not available for efficacy assessment. Additionally, the patient developed bone pain. Currently, it remains uncertain whether denosumab is ineffective for clinically diagnosed FD patients with GNAS-negative status. Therefore, comprehensive studies are needed to fully evaluate the safety and efficacy of denosumab in FD patients, particularly those with GNAS-negative mutations.

## 4. Conclusion

In conclusion, this is a 32-year-old woman with FD who experienced post-denosumab hypercalcemic rebound. While rebound hypercalcemia following denosumab cessation remains rare and predominantly affects adolescents, clinicians should implement mandatory calcium surveillance during treatment and post-therapy.

## Figures and Tables

**Table 1:** Previous cases of FD with denosumab.

No.	Reference	Patients (*n*)	Age (years)	Type of FD	Treatment	Rebound hypercalcemia
1	Boyce et al. [17]	1	9	MAS	1 mg/kg, every 3 M	Yes

2	Ganda and Seibel [18]	2	44 (case 1) 57 (case 2)	PFD (2)	60 mg, every 6–9 M (case 1), every 4 M (case 2)	No

3	Benhamou et al. [19]	1	48	FD	60 mg, every 6 M	Not available (NA)

4	Eller-Vainicher, 2016 [20]	1	20	MFD	60 mg, every 3 M	No

5	Majoor et al., 2019 [21]	12	38.3 ± 11.7	PFD (7) MAS (4) MFD (1)	60 mg, every 3–6 M	No

6	Gautam et al. [22]	1	45	PFD	60 mg (1 M), 120 mg (after 6 M, IY)	No

7	Meier et al. [23]	37	42 (19)	PFD (21) MAS (9) MFD (7)	60 mg, every 3–6 M	Yes (1)

8	Ikuta et al. [24]	1	27	MFD	120 mg, every 1 M, with loading doses on weeks 2 and 3 (after 3 M, 120 mg, every 2 M)	No

9	van der Bruggen et al. [12]	8	46.7 ± 14.7	PFD (5) MAS (2) MFD (1)	60–120 mg, every 3–6 M	NA

10	Raborn et al. 2021 [25]	1	13	MFD	53–70 mg, every 1–3 M	Yes

11	Meier et al. 2021 [26]	2	37 (case 1), 68 (case 2)	PFD (2)	60 mg, every 3 M	No

12	de Castro et al. [4]	8	29.9 ± 10.9	PFD (2) MAS (6)	120 mg, every 1 M, with loading doses on weeks 2 and 3	Yes (3)

13	Trojani et al. [27]	13	45.0 ± 14.0	PFD (4) MAS (5) MFD (4)	60–120 mg, every 2–6 M	No

14	Tucker-Bartley et al. [28]	1	24	MAS	30–60 mg, every 1 M	Yes

15	Abouammo et al. [29]	1	20	PFD	120 mg, every 1 M, with loading doses on weeks 2 and 3 (after 6 M, 60 mg, every 3 M)	No

## Data Availability

The data that support the findings of this study are available from the corresponding author upon reasonable request, and further details are sourced from the original articles cited.

## Nomenclature

FD: Fibrous dysplasia
RANKL: Receptor activator of nuclear factor kappa-B ligand
GNAS: G protein α-subunit
cAMP: Cyclic adenosine monophosphate
GCTB: Giant cell tumor of bone
CGCG: Central giant cell granuloma
ABC: Aneurysmal bone cyst.
